# Invasive ductal breast cancer metastatic to the sigmoid colon

**DOI:** 10.1186/1477-7819-10-256

**Published:** 2012-11-26

**Authors:** Xiao-cong Zhou, Hong Zhou, Ying-hai Ye, Xiu-feng Zhang, Yi Jiang

**Affiliations:** 1 Department of Surgery, The Dingli Clinical Institute of Wenzhou Medical College (Wenzhou Central Hospital), Wenzhou, Zhejiang, P.R. China; 2 Department of Colorectal Surgery, The Third People’s Hospital of Hangzhou, Hangzhou, Zhejiang, P.R. China; 3 Department of Pathology, The Dingli Clinical Institute of Wenzhou Medical College (Wenzhou Central Hospital), Wenzhou, Zhejiang, P.R. China

**Keywords:** Breast cancer, Sigmoid colon, Metastasis, Invasive ductal cancer

## Abstract

The most common sites of breast cancer metastasis are the bone, lung, liver and brain. However, colonic metastases from breast cancer are very rare in the clinic. We describe an unusual case of sigmoid colonic metastasis from invasive ductal breast cancer. With this report, we should increase the clinical awareness that any patient with a colorectal lesion and a history of malignancy should be considered to have a metastasis until proven otherwise. Early diagnosis is very important, which enables prompt initiation of systemic treatment, such as chemotherapy, endocrine therapy or both, thus avoiding unnecessary radical surgical resection and improving the prognosis.

## Background

Metastatic involvement of the gastrointestinal tract secondary to breast cancer is rare [1] and sigmoid colonic metastasis from breast cancer is even rarer. Metastatic breast carcinoma involvement of the gastrointestinal tract is usually of the lobular histologic subtype [2]. To the best of our knowledge, the case presented here is one very rarely reported in literature, showing sigmoid colonic metastasis from invasive ductal breast cancer.

## Case presentation

A 54-year-old woman was admitted to our hospital with pain in the left lower abdomen in April 2011. She had undergone modified radical mastectomy for an invasive ductal carcinoma of the right breast in December 2002. Histopathological examination revealed a 3.0 × 3.0 cm invasive ductal carcinoma of histological grade 3 and all seven resected axillary lymph nodes were negative for carcinoma. Immunohistochemistry (IHC) staining showed that the right breast cancer was positive for estrogen receptor (ER), progesterone receptor (PR) and p53, but negative for human epidermal growth factor receptor-2 (HER-2). The patient received adjuvant chemotherapy and endocrine therapy postoperatively. During a period of six years and one month, she was free of disease until January 2009, when she developed left breast carcinoma, for which she had undergone modified radical mastectomy. Histopathological examination revealed a 3.5 × 2.5 cm invasive ductal carcinoma of histological grade 3 and two of seventeen axillary lymph nodes were positive for carcinoma. IHC staining showed that the left breast cancer was positive for ER, PR and p53, but negative for HER-2. The patient received adjuvant chemotherapy, radiotherapy and endocrine therapy postoperatively. She had also undergone a total hysterectomy and bilateral salpingo-oophorectomy for left ovarian metastasis of breast carcinoma in November 2010, followed by second-line chemotherapy.

**Figure 1:**
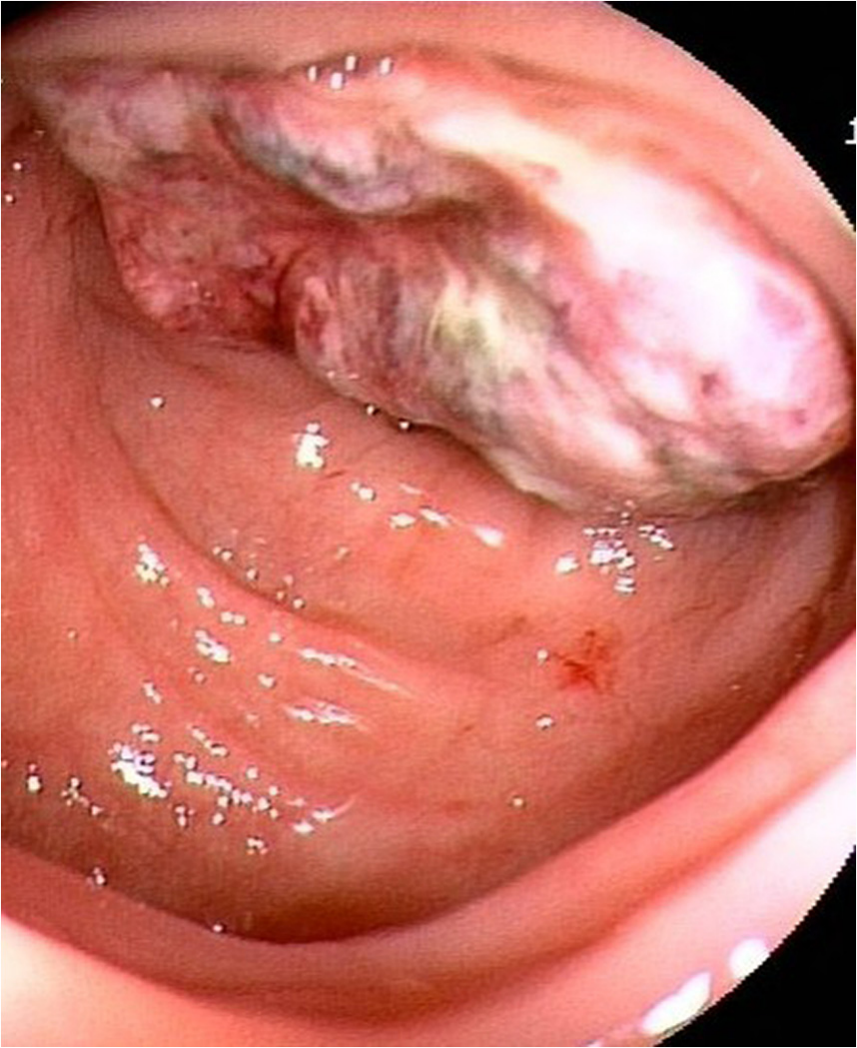
**Endoscopy of the sigmoid showed mucosal irregular hyperplasia at 16 to about 30 cm above the anal verge, taking up about half of the intestinal lumen.** The affected mucosal surface was eroded, necrotic, friable and prone to bleeding.

**Figure 2:**
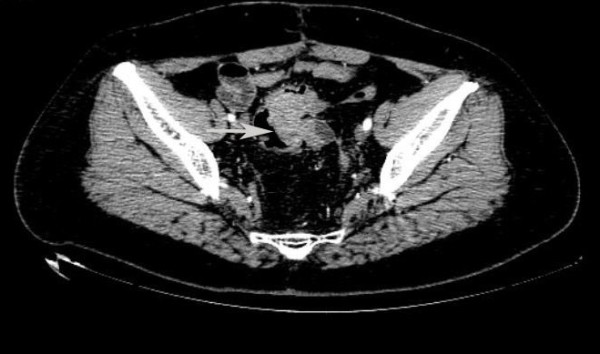
**Contrast-enhanced CT showed eccentric wall thickening of the distal sigmoid colon with a significantly enhanced soft tissue density mass causing an apparent stenosis (arrow) and nodular low density shadow in the left side.** CT, computed tomography.

**Figure 3:**
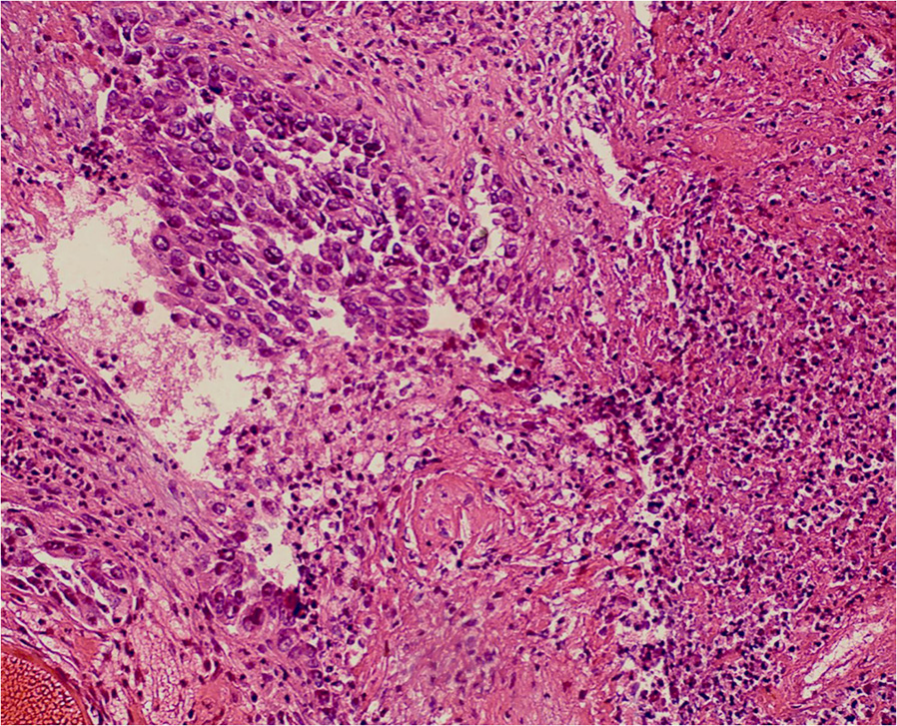
The sigmoid colonic biopsy specimen showed histological features of poorly differentiated adenocarcinoma which was quite similar to that of the previous invasive ductal breast cancer (hematoxylin&eosin stain; original magnification × 200).

**Figure 4:**
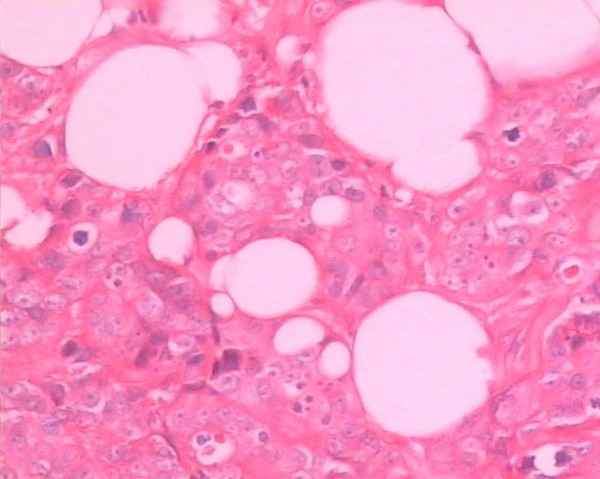
Invasive ductal carcinoma in the right breast (hematoxylin&eosin stain; original magnification × 400).

**Figure 5:**
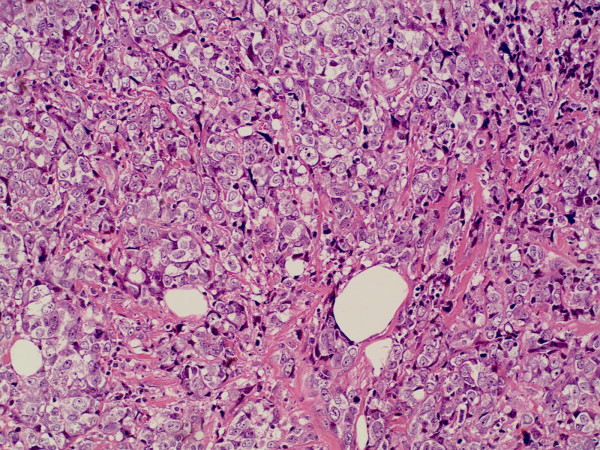
Invasive ductal carcinoma in the left breast (hematoxylin&eosin stain; original magnification × 200).

**Figure 6:**
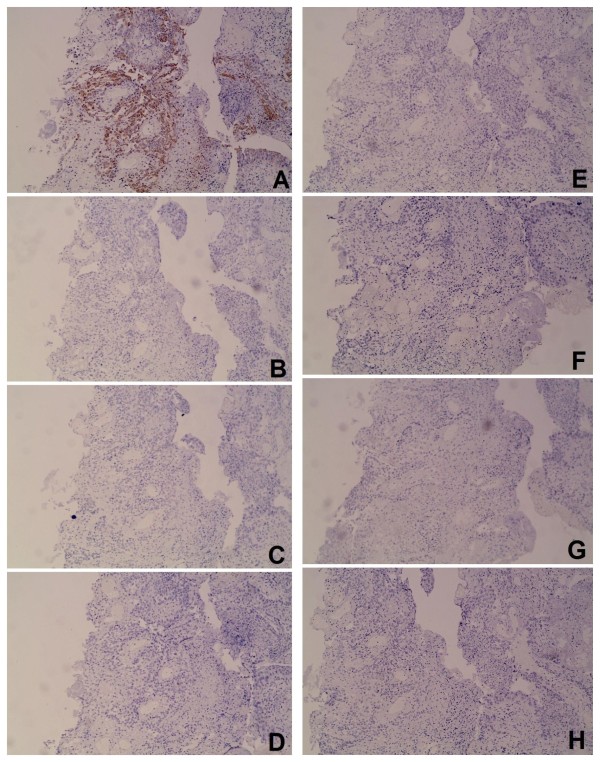
**Immunohistochemistry staining showed that the sigmoid colon cancer was positive for cytokeratin 7 (CK7) (original magnification × 100) (A).** Immunohistochemistry staining showed that the sigmoid colon cancer was negative for cytokeratin 20 (CK20)(original magnification × 100) (**B**). Immunohistochemistry staining showed that the sigmoid colon cancer was negative for cauda-related homeobox transcription factor 2 (CDX2)(original magnification × 100) (**C**). Immunohistochemistry staining showed that the sigmoid colon cancer was negative for Villin(original magnification × 100) (**D**). Immunohistochemistry staining showed that the sigmoid colon cancer was negative for thyroid transcription factor-1 (TTF-1) (original magnification × 100) (**E**). Immunohistochemistry staining showed that the sigmoid colon cancer was negative for human epidermal growth factor receptor-2 (HER-2)(original magnification × 100) (**F**). Immunohistochemistry staining showed that the sigmoid colon cancer was negative for estrogen receptor (ER)(original magnification × 100) (**G)**. Immunohistochemistry staining showed that the sigmoid colon cancer was negative for progesterone receptor (PR)(original magnification × 100) (**H**).

Physical examination revealed no palpable abdominal mass. Laboratory results showed mild anemia (hemoglobin level, 105 g/L), serum carbohydrate antigen (CA)125 level elevated to 134.4 kU/L (normal, 0 to about 35.0 kU/L), and serum CA724 level elevated to 139.8 kU/L (normal, 0 to about 6.9 kU/L), while serum levels of CA199, CA153, and carcinoembryonic antigen (CEA) were within the normal range. Endoscopy of the sigmoid showed mucosal irregular hyperplasia at 16 to about 30 cm above the anal verge, taking up about half of the intestinal lumen. The affected mucosal surface was eroded, necrotic, friable and prone to bleeding (Figure 1). Contrast-enhanced computed tomography (CT) showed eccentric wall thickening of the distal sigmoid colon with a significantly enhanced soft tissue density mass causing an apparent stenosis and nodular low density shadow in the left side (Figure 2). The sigmoid colonic biopsy specimen showed histological features of poorly differentiated adenocarcinoma (Figure 3) which was quite similar to that of the previous invasive ductal breast cancer (Figures 4 and 5). IHC staining (Figure 6) showed that the sigmoid colon cancer was negative for (cauda-related homeobox transcription factor 2) CDX2, Villin, thyroid transcription factor-1 (TTF-1), cytokeratin (CK)20, HER-2, ER and PR, but positive for CK7 and p53. IHC staining also showed that the positive cell population of Ki-67 was 30%. The sigmoid colonic lesion was, therefore, diagnosed to be a metastasis from the original breast cancer.

## Conclusions

The diagnosis was invasive ductal breast cancer metastatic to the sigmoid colon, which as a clinical entity can be easily misdiagnosed as primary sigmoid colon cancer. In the presented material, besides the medical history, a firm diagnosis of metastatic breast carcinoma to the sigmoid colon could be established fundamentally. It was based on the histology of the endoscopic biopsy specimen and immunohistochemistry. Metastatic breast carcinomas are often positive for CK7, ER and PR, but negative for CK20 and CDX2 [2]. CK7 was positive but CK20 and CDX2 were negative in the present case, which was similar to reports in the literature. However, ER and PR were negative in the sigmoid colon site, which was different from a positive expression of the primary bilateral invasive ductal breast cancer (Table 1). The conversion from hormone receptor positive in the primary tumor to hormone receptor negative in the metastasis has also been reported in a series of studies [3,4].

**Table 1:** Immunohistochemical markers for the primary bilateral invasive ductal breast cancer and metastases

	**The right breast cancer**	**The left breast cancer**	**The left ovarian metastasis**	**The sigmoid colonic metastasis**
ER	15% estrogen receptor positive	20% estrogen receptor positive	20% estrogen receptor weak positive	0 estrogen receptor positive
PR	10% progesterone receptor positive	5% progesterone receptor positive	0 progesterone receptor positive	0 progesterone receptor positive
HER-2	negative	negative	negative	negative
p53	positive	positive	positive	positive

The diagnosis of colorectal metastasis is difficult not only because it is infrequent but also because of its non-specific clinical presentation and variable radiographic features [5]. So clinicians should increase their clinical awareness that any patient with a colorectal lesion and a history of malignancy should be considered to have a metastasis until proven otherwise. The therapeutic management of colorectal metastasis is still controversial. Surgical resection should be reserved for palliation of intestinal obstruction or bleeding [6]. Many authors underline the importance of early diagnosis, which enables prompt initiation of systemic treatment, such as chemotherapy, endocrine therapy or both, thus avoiding unnecessary radical surgical resection and improving the prognosis. Our patient underwent second-line chemotherapy and endocrine therapy after the diagnosis of sigmoid colonic metastasis from breast cancer. She is alive with disease and in stable condition at present.

## Consent

Written informed consent was obtained from the patient for publication of this case report and any accompanying images.

## Abbreviations

CA: carbohydrate antigen; CDX2: cauda-related homeobox transcription factor 2; CEA: carcinoembryonic antigen; CK: cytokeratin; CT: computed tomography; ER: estrogen receptor; HER-2: human epidermal growth factor receptor-2; IHC: immunohistochemistry; PR: progesterone receptor; TTF-1: thyroid transcription factor-1.

## Competing interests

The authors declare that they have no competing interests.

## Authors’ contributions

XCZ performed the literature review, drafted and revised the manuscript. HZ, YHY and XFZ participated in the design of the study and revised the manuscript for intellectual content. YJ evaluated the histopathological features and contributed to the histopathological section of the manuscript. All authors read and approved the final manuscript.
